# Prevalence and pattern of multimorbidity among chronic kidney disease patients: a community study in chronic kidney disease hotspot area of Eastern India

**DOI:** 10.3389/fmed.2023.1131900

**Published:** 2023-05-12

**Authors:** Subrata Kumar Palo, Soumya Ranjan Nayak, Debadutta Sahoo, Swetalina Nayak, Ashis Kumar Mohapatra, Aviram Sahoo, Pujarini Dash, Sanghamitra Pati

**Affiliations:** ^1^Model Rural Health Research Unit, ICMR-RMRCBB, Cuttack, India; ^2^ICMR-Regional Medical Research Centre, Bhubaneswar, India

**Keywords:** CKD, multimorbidity, GFR, chronic conditions, Cramer’s coefficient

## Abstract

**Introduction:**

Chronic kidney disease (CKD) is mostly asymptomatic until reaching an advanced stage. Although conditions such as hypertension and diabetes can cause it, CKD can itself lead to secondary hypertension and cardiovascular disease (CVD). Understanding the types and prevalence of associated chronic conditions among CKD patient could help improve screening for early detection and case management.

**Methods:**

A cross sectional study of 252 CKD patients in Cuttack, Odisha (from the last 4 years CKD data base) was telephonically carried out using a validated Multimorbidity Assessment Questionnaire for Primary Care (MAQ-PC) tool with the help of an android Open Data Kit (ODK). Univariate descriptive analysis was done to determine the socio-demographic distribution of CKD patients. A Cramer’s heat map was generated for showing Cramer’s coefficient value of association of each diseases.

**Results:**

The mean age of participants was 54.11 (±11.5) years and 83.7% were male. Among the participants, 92.9% had chronic conditions (24.2% with one, 26.2% with two and 42.5% with three or more chronic conditions). Most prevalent chronic conditions were hypertension (48.4%), peptic ulcer disease (29.4%), osteoarthritis (27.8%) and diabetes (13.1%). Hypertension and osteoarthritis were found to be most commonly associated (Cramer’s V coefficient = 0.3).

**Conclusion:**

Increased vulnerability to chronic conditions among CKD patients make them at higher risk for mortality and compromised quality of life. Regular screening of CKD patient for other chronic conditions (hypertension, diabetes, peptic ulcer disease, osteoarthritis and heart diseases) would help in detecting them early and undertake prompt management. The existing national program could be leveraged to achieve this.

## Introduction

Chronic kidney disease (CKD) is defined as a decrease in Glomerular Filtration Rate (GFR) to less than 60 mL/min/1.73m^2^ for at least 3 months, irrespective of cause (1, 2). It was estimated, around 13% of the world’s population has CKD. Additionally, prevalence estimates imply the problem is 15% greater in low- and middle-income nations compared to high-income ones (3). CKD is mostly asymptomatic until its stage is advanced (4) and so, the documented cases presenting to hospitals represent the tip of the iceberg. As the disease stage progresses, it leads to many complications that include CVD (myocardial infarction, ischemic stroke, peripheral vascular disease, valvular disease and arrhythmias), anemia, bone mineral disease, volume overload and electrolyte imbalances (5). On the other hand, diseases such as hypertension and diabetes mellitus are known risk factors for CKD (6, 7). Hence, CKD is often linked to multimorbidity (3). Multimorbidity is defined as presence of two or more chronic conditions in the same individual at a point in time (8). Research study among older age group (>60 years) has shown multimorbidity to be prevalent in 73.9% of general participants and 86% among participants having CKD (any stage) (9). The presence of multimorbidity especially among CKD patients increases the risk of complications and influences the prognosis greatly leading to longer hospital stays, greater health-care expenses, mortality, polypharmacy and low quality of life (4, 10). Early detection of associated morbidities through detailed evaluation in the community and their prompt management will undoubtedly aid in preventing the potential consequences (10). As in other lower middle income countries (LMICs), multimorbidity has become the norm among Indian adults. Data on the prevalence and pattern of multimorbidity among CKD patients in India, particularly in a rural setting, are nonetheless lacking. Decision-making about the degree of multimorbidity in CKD patients in India is therefore hindered by contradicting clinical guidelines, particularly in a rural setting (11, 12). Because of this, contemporary health care programs urgently need guidance concerning the pattern and prevalence of multimorbidity.

In India, many states have hotspot regions for CKD including Odisha, an Eastern state (13). Badamba and Narasinghpur blocks of Cuttack district, Odisha, are in the limelight for CKD burden for last more than a decade and have brought the attention of researchers, program implementers and policy makers to prevent and control it (14). With this backdrop, a research study was carried out to determine prevalence of multimorbidity among CKD patients and explore the pattern of chronic conditions among CKD population of the said areas. This will primarily aid policy makers in identifying which other disease areas’ recommendations should be taken into account when incorporating renal disease recommendations. This study was done through Model Rural Health Research Unit (MRHRU) established in the catchment area of this CKD hotspot.

## Materials and methods

A cross-sectional study was conducted among the diagnosed CKD patient in the catchment area of MRHRU at Tigiria, a block of Cuttack district, Odisha. Under the study, a total of 674 CKD patients diagnosed between (January 2017–January 2021) were line listed after collecting data from the healthcare management information system (HMIS) database of community health centers of both the study blocks. Among all the patients, 97 were deceased, 99 were bed ridden and 221 could not be contacted (incorrect phone number). Finally, among the 257 CKD patients contacted for the study, 5 declined to participate, and a total of 252 interested participants who verbally consented for the study were enrolled, with a non-response rate of 1.95%.

### Data collection tool

A standardized structured schedule ‘Multimorbidity Assessment Questionnaire for Primary Care (MAQ-PC)’ was used for data collection. This is a validated tool for community based assessment of multimorbidity (15). Additionally, socio-demographic information was collected using a standardized tool. Considering the COVID-19 pandemic related restrictions during the study period, data were collected telephonically by the trained field investigators using an android based Open Data Kit (ODK) platform.

### Statistical analysis

Univariate descriptive analysis was done to determine the socio-demographic distribution of CKD patients tabulated by *n* (%) and confidence interval. To assess the distribution of associated disease with number of chronic conditions, a bivariate descriptive table was presented with *n* (%). A Cramer’s heat map was generated by estimating Cramer’s V coefficient value by using R studio 2021.09.1 to assess the association between diseases among participants having two or more chronic conditions other than CKD.

## Results

The mean age of the study participants was 54.11 (±11.57) years, ranging from20 to 86 years. While 52.4% were in the (40–59) age group, 37.7% were 60 or above and 9.9% were in the 20-39 age group. The majority (83.7%) were male, 97.6% of participants were Hindus and 91.5% were married. While most of the participants (88.2%) were from rural villages, 84.9% of participants were literate,69.1% of participants belonged to below poverty line (BPL) and 44.1% had monthly family income less than 6,000 Indian rupees. The detailed socio-demographic distribution of study participants is presented in Table 1.

**Table 1:** Distribution of participants according to socio-demographic characteristics.

Socio-demographic characteristics	Participant (*N* = 252) *n* (%)	95% Confidence interval
Age group
20–39	25 (9.92%)	(6.52–14.29%)
40–59	132 (52.38%)	(46.01–58.68%)
60 above	95 (37.70%)	(31.69–43.99%)
Gender
Male	211 (83.73%)	(23.25–78.58%)
Female	40 (15.94%)	(11.58–20.98%)
Religion
Hindu	245 (97.61%)	(94.36–98.87%)
Islam	6 (2.39%)	(0.87–5.11%)
Marital status
Currently married	226 (91.50%)	(87.29–94.66%)
Never married	11 (4.45%)	(2.24–7.82%)
Widow/Widower	10 (4.05%)	(1.95–7.31%)
Ethnicity
General	76 (30.77%)	(25.07–36.93%)
OBC	111 (44.94%)	(38.62–51.37%)
Other	60 (24.29%)	(19.07–30.13%)
Residence		
Urban	29 (11.84%)	(8.07–16.55%)
Rural	216 (88.16%)	(83.44–91.92%)
Education		
Illiterate	36 (15.13%)	(10.82–20.32%)
Literate	202 (84.87%)	(79.67–89.17%)
House type
Kutcha	58 (24.37%)	(19.05–30.33%)
Pucca	94 (39.50%)	(33.24–46.01%)
Semi pucca	86 (36.13%)	(30.02–42.59%)
Monthly family income
Below 6,000	101 (44.10%)	(37.56–50.79%)
Above 6,000	128 (55.90%)	(49.20–62.43%)
Poverty status
Above poverty line (APL)	73 (30.93%)	(25.09–37.25%)
Below poverty line (BPL)	163 (69.07%)	(62.74–74.90%)

**Figure 1:**
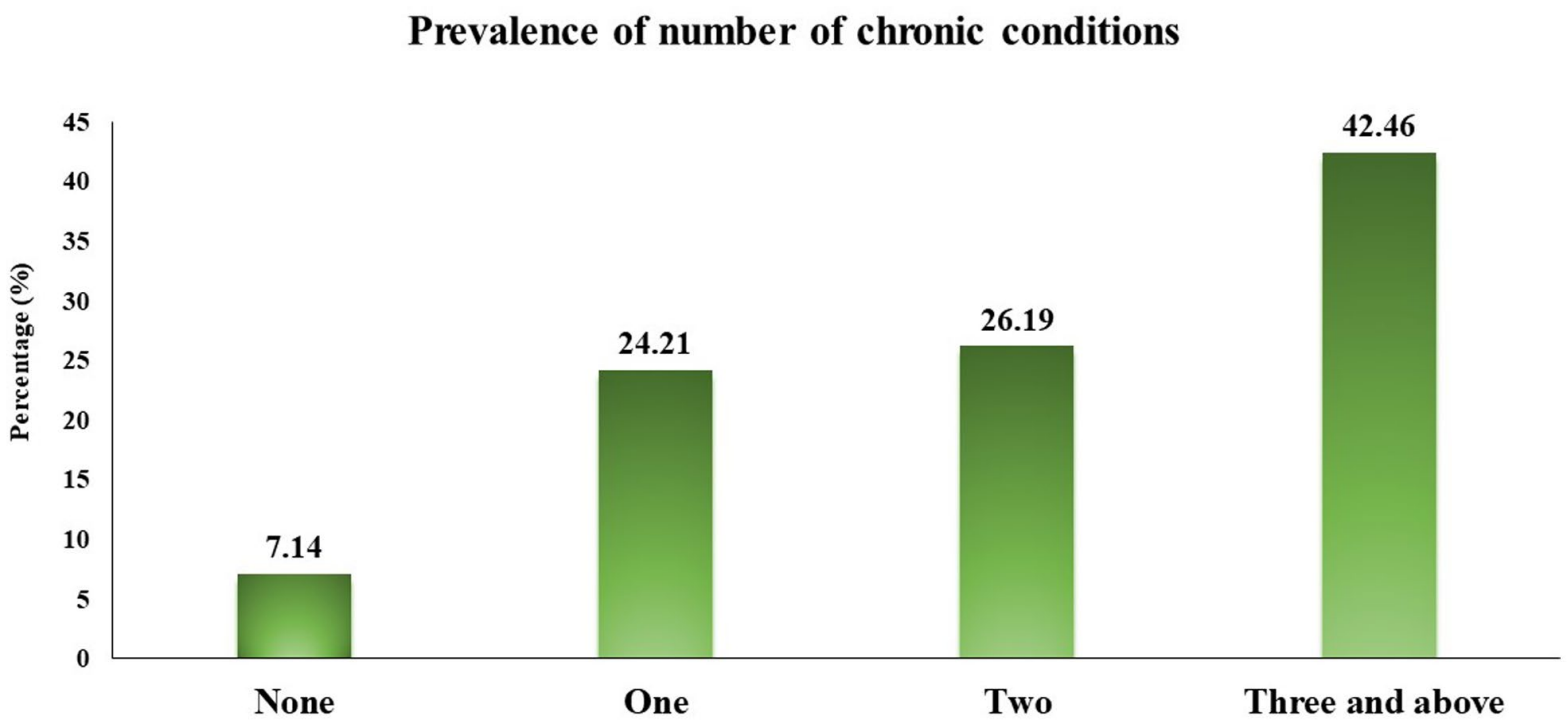
Distribution of number of other chronic conditions among participants.

Among 252 study participants, 234 (92.9%) had other chronic conditions apart from CKD (multimorbidity). Upon analyzing for the number of chronic conditions associated at the individual level, 18 (7.1%) had no other chronic condition, 61 (24.2%) had one additional chronic condition, 66 (26.2%) had two additional chronic conditions and 107 (42.5%) had three or more additional chronic conditions among CKD patients. The distribution of number of associated chronic conditions is presented in Figure 1.

**Figure 2:**
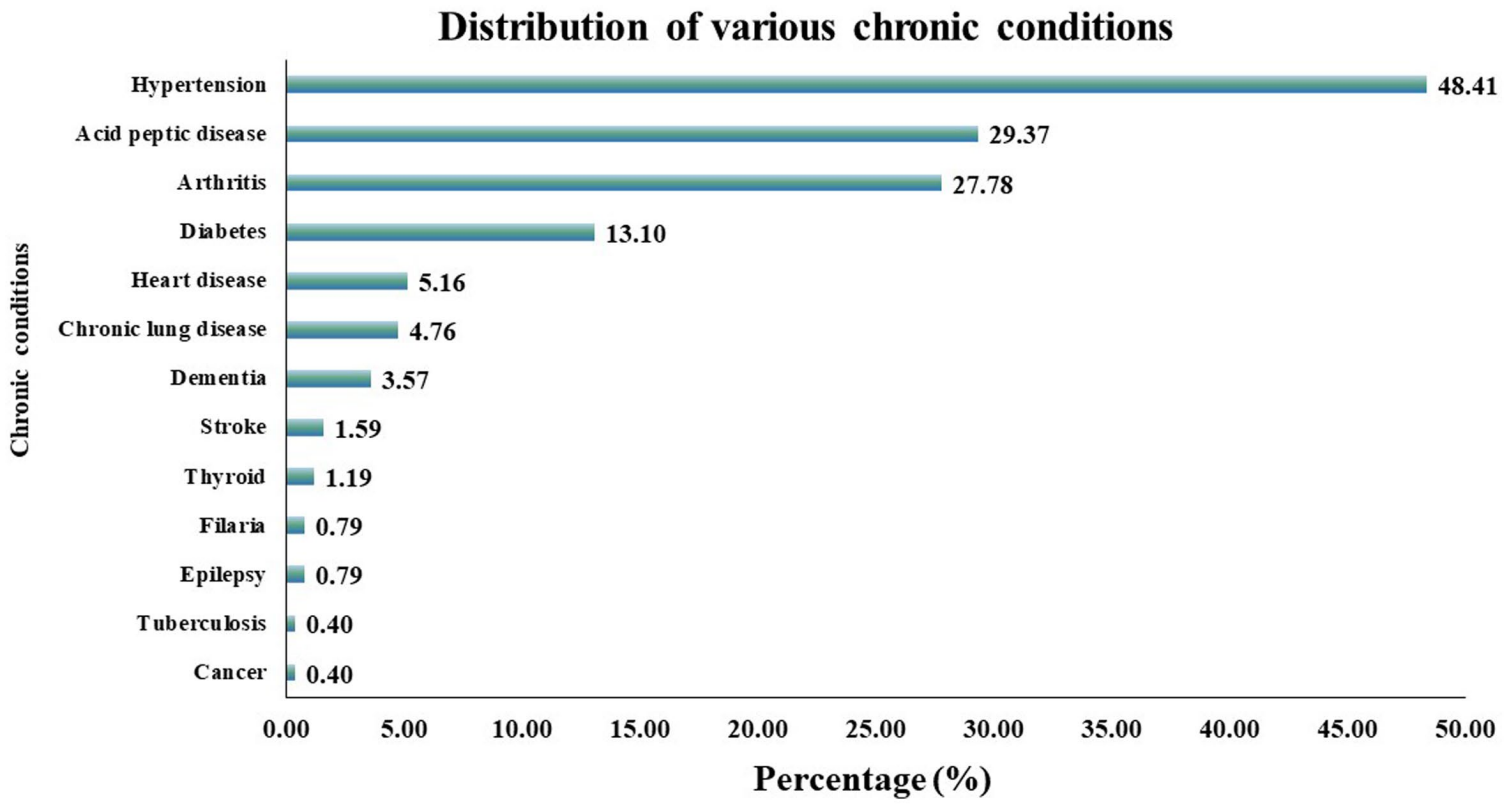
Prevalence of various chronic conditions among CKD participants with Multimorbidity.

Among the participants with multimorbidity, the major associated disease conditions included hypertension (48.41%), peptic ulcer disease (29.4%), osteoarthritis (27.8%) and diabetes mellitus (13.1%). The prevalence of different chronic conditions among CKD patients is depicted in Figure 2.

Upon further analysis of the associated morbidities according to number of chronic conditions among the participants with multimorbidity, a total of 346 co-morbid conditions were identified. Out of those, 15 (4.3%) were among participants having one additional chronic condition, 68 (19.6%) were among participants having two additional chronic conditions and 263 (76%) were among participants having three or more additional chronic conditions.

Among participants having one associated chronic condition, hypertension and peptic ulcer disease were the major associated conditions. Among participants with two associated morbidities, hypertension, osteoarthritis and peptic ulcer disease were the major conditions. Among participants with three or more additional comorbidities, hypertension, peptic ulcer disease, osteoarthritis and diabetes mellitus were the major associated conditions. The detailed distribution of chronic conditions is presented in Table 2.

**Table 2:** Distributions of number of associated disease conditions according to presence of number of chronic conditions.

Chronic conditions	None or One comorbidity	Two comorbidities	Three and above comorbidities
Hypertension	7 (46.7%)	28 (41.2%)	87 (33.1%)
Acid peptic	4 (26.7%)	11 (16.2%)	59 (22.4%)
Osteoarthritis	1 (6.7%)	21 (30.9%)	48 (18.2%)
Diabetes mellitus	2 (13.3%)	3 (4.4%)	28 (10.6%)
Heart disease	1 (6.7%)	1 (1.5%)	11 (4.2%)
Chronic lung disease	0	2 (2.9%)	10 (3.8%)
Dementia	0	0	9 (3.4%)
Stroke	0	0	4 (1.5%)
Thyroid	0	0	3 (1.1%)
Filaria	0	0	2 (0.8%)
Epilepsy	0	0	2 (0.8%)
Tuberculosis	0	1 (1.5%)	0
Cancer	0	1 (1.5%)	0
Total	15 (4.3%)	68 (19.7%)	263 (76%)

**Figure 3:**
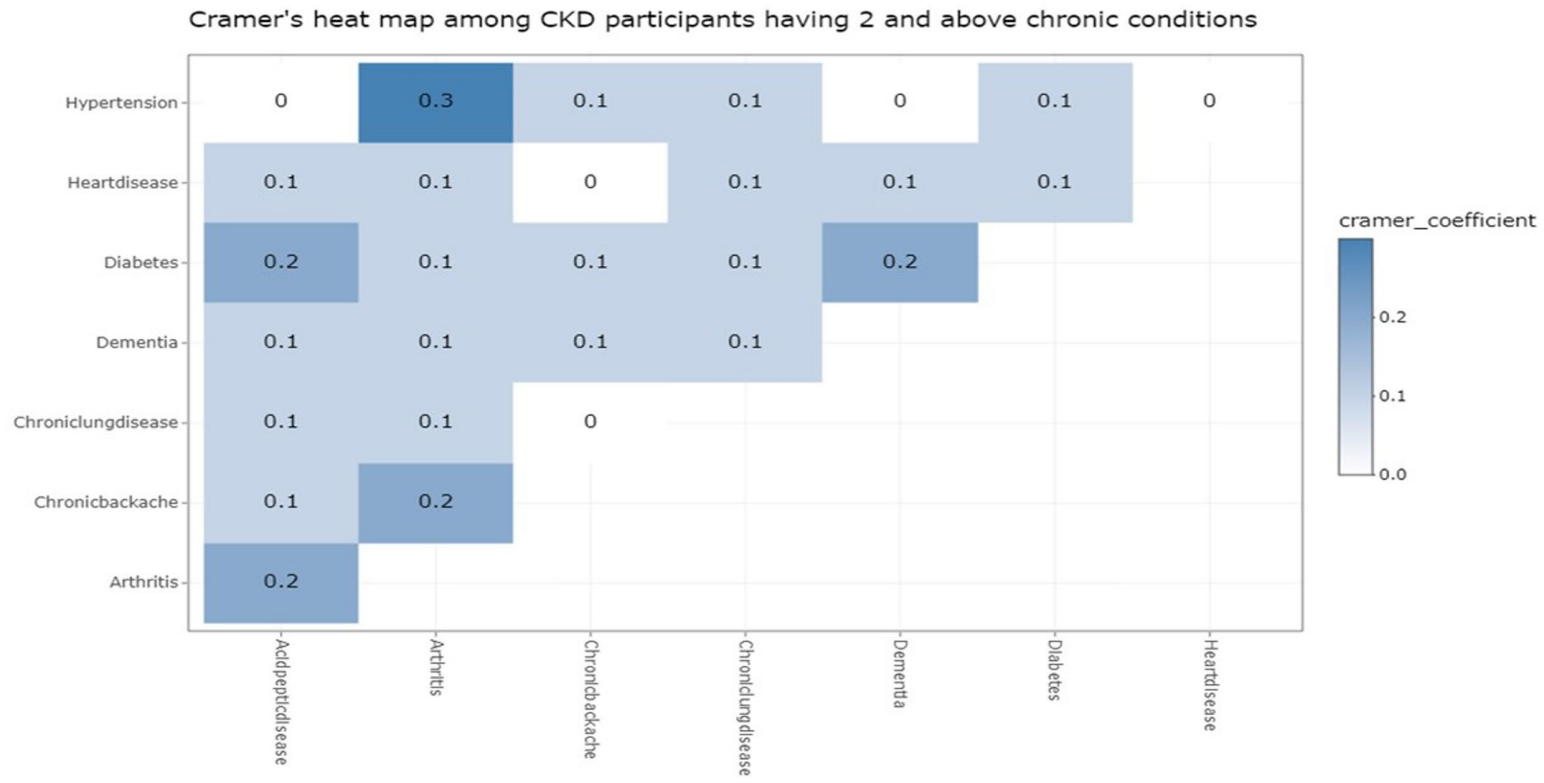
Crammer’s heat map showing the association between chronic conditions.

We further analyzed the co-occurrence of different diseases among the study participants having two and more additional chronic conditions. To find out this association between diseases, the eight most prevalent chronic conditions were considered to run a Cramer heat map (refer to Figure 2; Table 2). Cramer’s V coefficient lies between 0 and 1, where 0 indicates no association between diseases and 1 indicates a perfect strong association between the diseases. The highest crammer’s V coefficient value (0.3) was between hypertension and osteoarthritis. Crammer’s V coefficient value of 0.2 was observed between diabetes mellitus and peptic ulcer disease, chronic backache and osteoarthritis, osteoarthritis and peptic ulcer disease, and diabetes mellitus and dementia. Among other diseases, the Crammer’s V coefficient value was either 0.1 or 0. The association between morbidities is depicted in Figure 3 below.

## Discussion

With improved life expectancy, aging people are also at increased risk for chronic illnesses such as hypertension, diabetes mellitus, cancer, chronic kidney disease and mental health problems (16). In addition to aging, unhealthy lifestyles and unplanned exposure to urban environments attribute to the occurrence of non-communicable diseases (17). Due to limited research studies on multimorbidity among CKD patients, the present study has explored the prevalence of other chronic conditions among CKD patients in the hotspot area of Odisha, Eastern India. Additionally, we assessed the pattern of other chronic conditions among CKD patients.

CKD patients have a high prevalence of multimorbidity because CKD is a systemic disorder. In our study, we found that the majority of participants had two or more other chronic conditions in addition to CKD. This finding is supported by a study conducted in the UK with 1741 participants, which revealed that the majority of participants had two or more chronic conditions in addition to CKD (18). Our study also showed that about one-fifth of the participants had one or more other chronic conditions in addition to CKD, which is supported by a study among primary care patients that found 26 and 29% of all participants had one and two other chronic conditions, respectively, in addition to CKD (18). As the kidneys are responsible for filtering waste products, electrolyte balance, and important endocrine system functions, progression of CKD can cause toxin accumulation, electrolyte imbalances, and certain endocrine dysfunctions. As a result, CKD progression can cause disruptions to numerous metabolic pathways. While the sodium dysregulation, increased sympathetic nervous system, and alterations in the renin-angiotensin aldosterone system caused by CKD have primarily been associated with hypertension (19), these pathologic conditions could lead to the occurrence of other chronic diseases.

The presence of additional chronic conditions leads to an increased risk of mortality and a poor quality of life (18). The most prevalent associated disease conditions that we found were hypertension followed by peptic ulcer disease, osteoarthritis and diabetes mellitus. This finding was also reflected by a community based study from North Kerala which found the chronic conditions among CKD patient include hypertension (61.4%), diabetes (47.3%), cardiovascular disease (30.6%), chronic obstructive pulmonary disease (10%), malignancies (2.6%), and retinopathy (28%) (20). Both Studies found that hypertension is the most common co-morbid condition, and this correlates with the pathophysiology of CKD-associated hypertension where renin-angiotensin-aldosterone system (RAAS) over expression accompanied by eGFR reduction results in sodium and water retention (19).

Whereas CKD is an attributing factor for many other non-communicable diseases such as cardiovascular diseases, hypertension, anemia, bone mineral, volume overload and electrolyte imbalances (5), diseases such as hypertension and diabetes mellitus are risk factors for CKD (8). In addition, studies have shown that consumption of NSAIDs is another important CKD risk factor (21). Patients suffering from osteoarthritis often take pain medicines such as non-steroidal anti-inflammatory drugs (NSAIDs). Also, these drugs cause peptic ulcer diseases (22, 23). In this context, we also observed a higher association between hypertension and osteoarthritis, diabetes and peptic ulcer disease, and osteoarthritis and peptic ulcer disease. This clearly indicates that CKD patients are more vulnerable for having multimorbidity because of the risk factors involved, nature and complications of the disease, and the medications prescribed and consumed.

## Conclusion

In view of increased vulnerability for different chronic conditions among CKD patients leading to their higher risk of mortality and morbidity, it is critically important to regularly screen the CKD patient for hypertension, diabetes, peptic ulcer disease, osteoarthritis and heart diseases especially in CKD hotspot areas. Early detection of these chronic conditions and their prompt management could help in improving their quality of life and mortality. The current national program for prevention and control of cancer, diabetes mellitus, CVD and stroke (NPCDCS) through established health and wellness centers could be leveraged for this purpose.

## Data availability statement

The raw data supporting the conclusions of this article will be made available by the authors, without undue reservation.

## Ethics statement

The studies involving human participants were reviewed and approved by ICMR-Regional Medical Research Centre, Institutional Human Ethics Committee. Written informed consent to participate in this study was provided by the participants'.

## Author contributions

SKP, SP, DS, SN, and SRN: conceptualization, design of the study, acquisition, and writing original draft. SRN, DS, SN, AM, and AS: data collection, data curation, statistical analysis, and writing original draft. SKP, DS, PD, and SP: intellectual content, study supervision, critical review, and draft editing. SKP, SRN, DS, SN, AM, AS, PD, and SP: integrity of any part of the work are appropriately investigated and resolved. All authors approved the final draft.

## Conflict of interest

The authors declare that the research was conducted in the absence of any commercial or financial relationships that could be construed as a potential conflict of interest.

## Publisher’s note

All claims expressed in this article are solely those of the authors and do not necessarily represent those of their affiliated organizations, or those of the publisher, the editors and the reviewers. Any product that may be evaluated in this article, or claim that may be made by its manufacturer, is not guaranteed or endorsed by the publisher.
